# The Co-Relation of General and Dental Medicine and Surgery

**Published:** 1889-11

**Authors:** G. Frank Lydston

**Affiliations:** Chicago, Illinois


### COMMUNICATIONS.

### The Co-Relation of General and Dental Medicine and Surgery.

BY DR. G. FRANK LYDSTON, CHICAGO, ILLINOIS.

Lecturer on the Surgical Diseases of the Genito-Urinary Organs, and Venereal Diseases, in the Chicago College of Physicians and Surgeons, Chicago, Ill.

I shall endeavor in this article to present my reasons for believing that the interests of the general practitioner and dentist have much in common. Personally, I am greatly interested in dentistry as a department of the great science and art of general medicine, and in my association with dentists I have derived both social and professional benefits, and have come to regard them as the co-laborers of their medical and surgical brethren in the field of science. It was long argued by the members of my own department of science that the normal position of the dentist is that of a species of tooth-carpenter, a mere mechanical drawer of teeth and plugger of real or imaginary cavities, and it is a serious reflection upon the liberality of the medical profession that many of its members accord much the same position to the dentist of to-day.

In not a few instances the modern surgeon has indignantly resented the claims of dentistry as a surgical specialty, apparently forgetting in his illiberality, that it was only about a hundred years ago that the general surgeon himself began to rise above the level of the barber and the monkish cutter for "the stone." Had there not been a Hunter, who arose like an apostle of scientific light, and began his physio-chirurgical experiments years in advance of his day and age, the present status of general surgery might be most humiliating to contemplate.

It is an amusing fact that in some of our latter-day illustrations of the crudity of the surgery of a past generation, dentistry and general surgery are most intimately associated. How consoling and gratifying to the conceit of the pompous surgeon of to-day is the legend still to be occasionally seen surmounting a barber's pole, "bleeding, leeching, cupping, blistering, tooth-drawing, and hair-cutting done here."

Wedded centuries ago, surgery and dentistry drifted away from the barber, and unfortunately also from each other, *pari passu* with scientific progress. In these modern times we are striving to reunite the two branches of healing by a common interest in science, and upon the basis of a division of labor, *i. e.*, specialism. It is perhaps fortunate for all concerned that an occasional barber exists who is capable of demonstrating to us the common and humble origin of both specialties.

There has been a striking inter-dependence of most of the modern improvements in dental and surgical science. Although not generally so accepted, John Hunter's famous experiment of grafting the spur of a chicken cock upon its comb, really foreshadowed, not only the operation of skin-grafting, but that of tooth-grafting and tooth-implantation; a procedure which has been brought so prominently before the dental profession by Dr. Younger. It is indeed singular that the operations of tooth-transplantation and implantation was not earlier discovered, inasmuch as the various epithelial tissues and appendages are much alike in their manner of growth and development, and the growth of an implanted or transplanted tooth is hardly more remarkable than the phonomena attendant upon the attachment and growth of miliary skin grafts. That a tooth which has been apparently dead for a considerable time should grow after implantation is no more wonderful than the growth of skin grafts which have been transplanted from the dead to the living body. I have, myself, succeeded in the transplantation of grafts from a corpse, dead forty hours, to a healthy living ulcer. As an absolute verification of this, I have grafted the skin of a negro, dead nearly thirty-six hours, to the leg of a white man, and with success, the area of black skin resulting being quite conclusive. Another peculiar fact is that apparently effete epithelium scraped from the surface of the skin, and sprinkled over the granulations of a healthy ulcer, will result in the development of new little islets of skin and thus materially hasten cicatrization.

I understand that living teeth have been transferred from the human mouth to the comb of a cock, and thus preserved for future use, the teeth meanwhile absorbing nutriment from the circulation of the fowl. If this is practicable, the procedure brings dental surgery of to-day, and the now famous experiment of the immortal Hunter, very near each other. While my digression may be irrelevant, I think that it is justified by this kinship between skin-grafting and tooth-grafting.

The histo-genetic power shown to exist in epithelial structures has been shown to exist in others. There seems to be a general law pervading all organic life, to the effect that the power of reproduction and repair of organized matter is inverse ratio to its degree of differentiation. This holds true of cells, as well as bodies completely organized. The star-fish may be dismembered, but it reproduces the lost segments. Smash the amœba, and we have as many amœbæ as we have particles. Spiders, crabs, cray fishes, and some other animals, speedily reproduce lost limbs and other appendages. So we see that epithelial cells are possessed of great vitality, and not only grow themselves, but excite the property of rapid growth in any cell with which they may come in contact. Degraded epithelial cells grow more rapidly than the more perfect forms, as is evidenced by those sad cases of carcinoma and sarcoma so often met with. Connective tissue cells have an innate power of proliferation and propensity for differentiation, upon which all processes of repair and growth of all kinds, physiological and pathological, depend. Irritate the tissues and these propensities are forthwith brought into action.

The labors of the general surgeon, showed us long ago, that bone could be reproduced from periosteum, and that most daring operator, the late James R. Wood, showed in his noted case of phosphorus necrosis, that the entire lower jaw might be reproduced after its exsection.

The dental and oral surgeon has not been at all backward in taking advantage of this extraordinary reproductive power of the maxillary periosteum, and my progressive friend, Dr. J. S. Marshall, has operated upon the maxilla in a manner which would put some of our general surgeons to the blush. Such operations do not savor very strongly of the tooth-pulling machanic.

The dentist has not only shown himself to be capable of assimilating the discoveries of surgical science, but he has contributed materially to the art of surgery. To a dentist we owe the discovery of anæsthesia, the greatest boon ever conferred upon suffering humanity. Much of our knowledge of that valuable drug, the peroxide of hydrogen, has been due to its use in the practice of dentistry. Even the field of microscopy has been invaded by the dental scientist. Much of our present knowledge of the forms and growth of micro-organisms is due to the researches of that indefatigable worker, Dr. G. V. Black, and it would be well if every physician would study the results of this gentleman's labor in his chosen field.

We have not only come to regard dentistɹy as a special branch of the healing art, but we are forced to acknowledge that it is a most important branch.

In the words of the late Professor Gross, "Dentistry is the most important specialty in medicine. Many people come into the world and go out of it who never require the services of other specialists, but no child is born that does not, sooner or later, require the services of the dentist." The words of the Nestor of American surgery are excellent testimony in favor of dentistry as a necessary specialty, and it would be well if their truth were more generally apprciated.

The classification of dentistry as a specialty of general surgery is justified to a greater degree than in the case of almost any other specialty. As my lamented friend, the late Professor Van Buren, used to define it, "Surgery is that branch of the healing art which takes charge of all those diseases and injuries which require, in their management, the use of instruments or mechanical appliances, operations, or especial manual dexterity." To no other specialty does this definition so aptly apply as to dentistry. I can scarcely conceive of a single dental or oral operation that does not require all of the qualifications mentioned. This eminent surgeon said further, with reference to general surgery as a specialty of medical science, "That a specialty should be the outgrowth of a liberal professional education," in short, that a true specialist should be a man who knows "something of everything, and everything of something." Every specialty should be evolved from the general field of scientific labor, and its selection should be modified by experience, the law of division of labor, and special adaptation. In the present article we have but to do with the dental specialist, and it is to him in particular that I at present apply this general rule.

The dentist nowadays is expected to be a fair anatomist, and it should no longer be considered reprehensible for him to know more than the anatomy of the teeth and jaws. He may never realize the fact that he has an ileo-caecal valve, or a vermiform appendix, but a little enlightenment upon the subject will not render him less capable of skillful dental work, and when an obstreperous cherry-pit lodges in his appendix, he may console himself in the fact that he knows pretty nearly where the trouble is located. He is, of course, expected to know all about the structure and development of the teeth and jaws, the muscular mechanics involved in their movements, and their nervous and vascular supply, in order that he may, on occasions, discourse learnedly upon centers of ossification, intermaxillary bones, and synchondroses *ad infinitum*. The necessity for a knowledge of physiology is self-evident, embryology, development and eruption of the teeth being of especial importance. A knowledge of the physiological secretions is a necessity, else how could the effects upon the teeth of the various morbid conditions affecting them be studied? In the study of normal and morbid secretions a knowledge of chemistry is necessarily involved. Chemistry and metallurgy are further necessary in order that the student of dental science may become familiar with the various materials and implements with which he works, and the composition of the teeth. The dentist requires a certain amount of therapeutical and pharmaceutical information. The dental materia medica is becoming an important factor in dental education. Perhaps the most important branch in dental education is pathology and pathological anatomy, particularly that division of the subject which has been termed "surgical pathology." The amount of general medical and surgical knowledge requisite to a clear understanding of the morbid changes which are brought under the attention of the dentist, is far greater than is supposed by those narrow-minded individuals who regard dentistry as a species of tooth-carpentry. The dentist must thoroughly understand the forms, causes, and treatment of caries and necrosis of the teeth and jaws, a knowledge of which will open up to him a vast field of general surgical information. If he understands maxillary necrosis he will also understand the same disease as affecting other bones. He should pay considerable attention to syphilis and its treatment, as causes of caries and necrosis of the teeth and jaws, and inflammation, or ulceration of the mucous membrane of the mouth. Being familiar with such lesions, he must necessarily know much of the character of mucous inflammation and ulceration in other situations. He must learn all about the causes, anatomy, and treatment of suppurative inflammation about the jaws, and when he has done so, he will have mastered the most important portion of general surgery.

Neoplasmata, their causee, method of formation and structure, should be familiar to him, for he may be confronted at any time with epulis, cancer of the jaws, exostoses, or other conditions requiring surgical skill for their relief or cure. He should know all of the important details regarding fractures, their method of union, and mechanical and therapeutical treatment. The necessity of a thorough understanding of the general principles of medicine, particularly as regards the causes, results and therapeutics of perversions of general nutrition, can scarcely be overestimated. This is most aptly illustrated in those conditions of malnutrition which result from syphilis, rickets, and struma, diseases which react most injuriously upon the teeth and jaws. A fair ability to differentiate the various oral lesions of syphilis from benignant affections, and from each other, is requisite.

It is a long way around from defective molars to perverted nutrition, but from imperfect mastication may result imperfect digestion and assimilation, and from the latter condition there may occur such a strain upon the liver and kidneys that actual structural disease results. Gout is a dyscrasic affection which may be expected from such conditions of perverted physiology. Conversely, as has been already indicated, morbid appearances of the teeth and gums may indicate certain morbid conditions of the general system, which bear a causal relation to the dental imperfections. As illustrations, may be mentioned syphilis, rickets, mercurialism, struma, and scurvy.

A subject with which every dentist should be more or less familiar is the serious affection known as septicæmia or blood poisoning. Instances of fatal blood poisoning have been known to follow so simple an affection as alveolar abscess, as well as more grave forms of suppurative inflammation about the jaws and teeth. Mild cases are more frequent than is generally supposed. Only a short time since I met with a case of mild septæmia following an alveolar abscess in one of my personal friends. In this instance the dentist in attendance, and a physician to whom he referred the patient prior to his visit to me, failed to recognize the real condition.

In connection with this condition of sepsis the dentist may simplify his studies of the subject if he will remember that septicæmia and pyæmia so-called, are but phases of the same constitutional condition, and are due to the local absorption and subsequent general dissemination of the same septic material. The differences between the two phases of disease are those of degree, not kind, and they depend, not upon the existence of a different *materies morbi*, but upon (1) the primary intensity of the poison; (2) the facility with which it is absorbed; (3) the quantity absorbed, and the duration of the period of its absorption; (4) the rapidity of its elimination; (5) and the most important consideration of all, the inherent vitality or resisting power of the patient.

By studying the subject of blood poisoning in this manner, it may be reduced to a simple and logical basis. As presented in most of the works on surgery, septicæmia and its various phases constitute a subject so confusing that very few students ever succeed in mastering its intricacies. The dental specialist should not only familiarize himself with the clinical features of septic infection, but he should study the results obtained by the investigations of Pasteur, which foreshadowed all our knowledge of septic processes. He should also understand the principles laid down by Lister in his system of antiseptic treatment, which involves all of those cardinal principles which govern us in the prophylaxis and treatment of blood infection from suppurating wounds. Look where we may in the chosen field of the dentist, and we are confronted by intricate problems of a general and most diverse surgical character. Who among our general surgeons can say after such a survey of the science and art of dental and oral surgery, that the skillful and studious practitioner of this specialty, is not a surgical scientist and a co-laborer in that beneficent art whose progress has been illumined by Hunter, Billroth, Paget, Pare, Mott, Van Buren, Wood, and others whose names, though equally famous, would fill my paper to overflowing. I maintain that no man, of even average intelligence, can become a thorough dentist without at the same time becoming a pretty good physician and surgeon. He should by all means possess the general knowledge of the average practitioner of general medicine, and in addition should have the special knowledge and mechanical talent necessary to treat the peculiar class of cases brought before him as a surgical specialist.

There is a special necessity for general medical knowledge in a certain direction, to which I desire to call attention. Every educated dentist is now supposed to familiarize himself with anæsthetics and their methods of administration, but unfortunately he is not expected to know very much about those conditions which contra-indicate their use. There are various morbid conditions of the heart, lungs and kidneys which the merest tyro might discover by a little attention, and which, I fear, often escape the attention of the dentist. I believe that no one should ever give an anæsthetic who is not qualified to make a fairly accurate estimate of the condition of the heart, lungs and kidneys, yet this is often done in the dentist's chair. Much can be determined by careful observation of the patient's general appearance. I recall several cases in point. A friend of mine, and a competent dentist, undertook the administration of chloroform for the purpose of extracting a carious tooth. He was assisted by a lady physician who considered herself well qualified. The affair did not concern me as I had an adjoining office, but I happened to get a glimpse of the patient's face and decided to remain within call. Hardly had the anæsthetic fairly begun its work before the stertorous breathing of the patient alarmed my friend so that he called me to his assistance. It was high time, for if ever a patient was on the verge of death from asphyxia and heart paralysis, this one was. I succeeded in resuscitating her, but she subsequently passed through an attack of renal and pulmonary congestion that well nigh ended her life. The puffy eye lids, red face, and feeble circulation of this patient should have warned my friend of the danger of vaso-motor paresis to which this patient was exposed, and should have suggested an urinalysis which would have developed the existence of chronic Bright's disease. I have since seen a similar case from nitrous oxide. In this case also there was the sequence of acute pulmonary congestion, but in a patient who was phthisical. There is an old adage to the effect that "it is a poor rule that does not work both ways." The doctor should have some special dental knowledge.

Some time ago I was consulted by a young man with a suppuarting tumor of the jaw. He had been treated for some months by an aspiring surgeon who pronounced the case one of *actinomycosis* and had proposed removal of a section of the jaw. Upon examination I found the case to be simply one of chronic phlegmonous inflammation of the tissues beneath the angle of the jaw, the process being secondary to carious teeth which the young man stated had been troubling him for some months. The tooth was extracted and the trouble subsided completely within two weeks. Incidentally I would state that this same surgeon made a report of a case which he claimed was precisely similar to the one which came under my observation. In this case, however, he removed a portion of the jaw, and strange to state, found and removed three carious teeth after the more serious operation had been performed. These teeth, he claimed, were the port of entry of the actinomycosis. The very pertinent question suggests itself in this connection, would not extraction of those carious teeth have been likely to spoil this operation for a rare and interesting disease? I trust that the time will arrive when the merest tyro in medicine and surgery will be sufficiently familiar with oral and dental lesions to avoid such serious mistakes as this. An interesting case showing the importance of a knowledge of the surgery of the mouth was referred to me a short time ago by a physician of this city. This case had been operated upon several times and a portion of the affected gum removed. On examination I found a spongy, vascular growth the size of a hickory nut protruding upon both the anterior and posterior surfaces of the lower incisor teeth. On examination I discovered the tumor to be a mass of spongy and exuberant granulations due to the irritation produced by a mass of tartar about the incisors. Removal of the tartar with the application of nitrate of silver to the tumor cured the case completely.

A fact recently determined and one which is of great importance, is the association of neuralgias of various kinds and perturbations of sight and hearing with carious teeth. This necessitated a certain amount of neurological knowledge on the part of the dentist. Without this knowledge he is apt to persistently treat or to fill teeth, the irritation produced by which is responsible for serious reflex nervous phenomena. Such ill-timed conservatism is quite as reprehensible as the opposite extreme of indiscriminate extraction of offending teeth. Not only is a knowledge of these reflex phenomena necessary on the part of the dentist, but the physician himself should be thoroughly posted upon the inter-communication and co-relation of nervous supply of the various parts about the head and organs of special sense. It is very humiliating indeed to have a patient whom we have treated for months for an obstinate cephalalgia, neuralgia, or otalgia, determine upon his own responsibility that the offending tooth is the cause of the difficulty, go to the dentist, have it extracted, and immediately recover. Such humiliating cases are in my opinion, frequent experiences in the practice of the majority of physicians. I am free to say that such cases have come under my own experience.

Dr. Samuel Sexton, of New York, has written some very elaborate dissertations upon the co-relation of dental and oral lesions with disturbance of the auditory apparatus. Other writers have given equal attention to ocular disturbance incident to the same processes. The most frequent condition to be met with, dependent upon carious teeth, is trigeminal neuralgia. This illustration is so familiar to both dentist and physician that it would seem hardly necessary for me to expatiate upon it, were it not for the fact that cases of mistaken diagnosis are constantly occuring. It is my opinion that hundreds of unfortunate individuals are yearly drugged and dosed with powerful remedies for the relief of neuralgic affections which are directly dependent upon causes that the simple operation of extraction of the carious teeth would readily remove.

The association of causes of direct irritation in parts remote from the affected nerve filaments is just attracting its meed of attention on the part of the profession. It is found, for example, that certain lesions of the nose are responsible for many cases of asthma which were formerly supposed to be essential and dependent upon causes intrinsic of the pulmonary tissue or bronchioles, or even ascribed to such obscure causes as indigestion, cardiac insufficiency, rheumatism, etc. Certain cases of so general and marked constitutional disturbance as hay fever have been relieved by the destruction of certain sensitive areas in the tissues covering the turbinated bones of the nares. Cases of obstinate and intractable cough which have been treated for years by multitudinous remedies and divers physicians, much to the detriment of the patient, but with little disturbance to the condition for which he has been treated, have been speedily and permanently cured by the removal of certain causes of local irritation in the throat, nose, or external auditory meatus.

In passing I will merely allude to the necessity for a knowledge of some of the natural sequences of pregnancy. The better class of dentists are of course familiar with the fact that dental operations are not always attended by good results, and are sometimes absolutely dangerous in the pregnant state. This fact, although disputed by some, is of sufficient importance to merit most serious consideration. Even if disputed, the patient and not the operation, should have the benefit of the doubt.

